# Risk factors of additional surgery after non-curative endoscopic submucosal dissection for early gastric cancer

**DOI:** 10.1186/s12876-023-03006-9

**Published:** 2023-11-10

**Authors:** Feng Sun, Yibo Huang, Yan Sun, Xingzhou Wang, Shichao Ai, Wenxian Guan, Meng Wang

**Affiliations:** 1 Division of Gastric Surgery, Department of General Surgery, Nanjing Drum Tower Hospital, the Affiliated Hospital of Medical School, Nanjing University, Nanjing, China; 2 Division of Gastric Surgery, Department of General Surgery, Nanjing Drum Tower Hospital Clinical College of Nanjing Medical University, Nanjing, China; 3 Department of Anesthesiology, Nanjing Drum Tower Hospital, the Affiliated Hospital of Medical School, Nanjing University, Nanjing, China

**Keywords:** Early gastric cancer, Endoscopic submucosal dissection, Additional Surgery, Residual cancer, Lymph node Metastasis, Histologic type

## Abstract

**Background:**

The criteria for surgical intervention after non-curative endoscopic submucosal dissection (ESD) of early gastric cancer are unclear. We aimed to clarify the risk factors for residual cancer and lymph node metastasis after non-curative ESD and to identify recommendations for additional surgery.

**Methods:**

We collected data on 133 consecutive patients who underwent additional surgery after non-curative ESD of early gastric cancer at Nanjing Drum Tower Hospital from January 2013 to July 2022. Univariate and multivariate analyses were performed to seek risk factors of residual cancer and lymph node metastasis.

**Results:**

The incidence rates of residual cancer and lymph node metastasis were 13.5% (18/133) and 10.5% (14/133), respectively. There was neither residual tumor nor lymph node metastasis in 104 (78.2%) cases. Multivariate analyses elucidated that horizontal margin was an independent risk factor for local residual cancer, whereas lymphatic infiltration was an independent risk factor for lymph node metastasis. Patients with mixed histological types were more likely to suffer lymph node metastasis and further undergo additional surgery after non-curative ESD than pure histological type.

**Conclusions:**

Additional gastrectomy with lymph node dissection was strongly recommended in patients with lymphatic infiltration after non-curative ESD of early gastric cancer. Patients with mixed histological type have a high propensity for lymph node metastasis and should be treated as a separate subtype.

**Supplementary Information:**

The online version contains supplementary material available at 10.1186/s12876-023-03006-9.

## Introduction

With the popularization of endoscopic technology and early cancer screening, the proportion of early gastric cancer diagnosed is increasing. At present, endoscopic mucosal resection (EMR) and endoscopic submucosal dissection (ESD) are fundamental treatments for early gastric cancer (EGC). Meanwhile, various large multi-center randomized controlled trials were carried out to expand the indications of endoscopic therapy. An important issue would ensue inevitably that a subset of patients might undergo non-curative resection.

According to the Guidelines for endoscopic submucosal dissection and endoscopic mucosal resection for early gastric cancer updated in 2020 [1], the curable resection grades were divided into eCuraA, eCuraB, and eCuraC (Supplemental Table 1). eCura C was defined as the noncurative resection. eCuraC was further divided into eCuraC-1 and eCuraC-2, which represents non-en bloc resection or positive horizontal margins, and adjoint of high-risk factors for lymph node metastasis, respectively. For eCuraC-1 lesions, additional ESD, close follow-up, or additional surgery is optional after adequate communication with the patient [2, 3]. For eCuraC-2 lesions, gastrectomy with lymph node dissection is recommended [4].

In clinical work, it is common that neither residual tumor nor lymph node metastasis was found after additional surgery. In addition, there are some patients who hesitate to choose the following measures after non-curative ESD. Therefore, it is necessary to access accurately the risk of residual cancer and lymph node metastasis after ESD, which was fundamental for the choice of re-medical measures after non-curative ESD. In this study, we collected cases of additional surgery after non-curative ESD and analyzed the risk factors for residual cancer and lymph node metastasis.

## Methods

### Patients

133 consecutive patients who underwent additional surgery after non-curative ESD at Nanjing Drum Tower Hospital from January 2013 to July 2022 were analyzed in this study. All patients had pathologically confirmed early gastric cancer. Patients with synchronous other malignant tumors, previous stomach surgery, or incomplete clinical data were excluded. Patients were followed-up by phone call or outpatient clinic at 3 months after surgery and once a year thereafter. The follow-up deadline was 2023-09-15. This study was approved by the hospital ethical committee of Nanjing Drum Tower Hospital.

### Data collection

The collected general data include age and gender. The collected pathological data consisted of tumor location, lesion size, differentiation type, depth of invasion, ulceration, lymphovascular invasion, perineural infiltration, and lesion margin. For differentiation type, we defined the mixed histologic type as that consisting of both differentiated and undifferentiated lesions. Postoperative characteristics included short-term complications and long-term follow-up. The postoperative short-term complications were defined as morbidity or mortality that occurred during hospitalization or within 30 days after surgery.

### Non-curative criteria

The ESD indication complied with the Japanese gastric cancer treatment guidelines. The infiltration depth of early gastric cancer was divided into mucosal (T1a) and submucosal (T1b). Submucosal lesions were further classified as superficial (depth < 500 μm; SM1) and deep (depth ≥ 500 μm; SM2) submucosal infiltration. The non-curative criteria of ESD satisfied one of the following items: non-en bloc resection, positive margins, lymphovascular infiltration, SM2 submucosal invasion, differentiated type (diameter > 3 cm) with ulceration or SM1 submucosal invasion; undifferentiated type with submucosal invasion or ulceration or diameter > 2 cm.

### Statistical analysis

The categorical variables were presented as numbers. Differences between categorical variables were compared with the Chi-squared test or Fisher exact test. Univariate analyses were performed first to seek risk factors of residual cancer and lymph node metastasis. Further multivariate analyses were performed on variables that were statistically significant in univariate analyses. Kaplan-Meier curves and the log-rank test was conducted to compare the long-term outcome. SPSS 19.0 (Chicago, IL, USA) was used for all statistical analyses. Statistical differences were set at P value < 0.05.

## Results

### Patient characteristics

As shown in Table 1, a total of 133 patients who underwent additional surgery after non-curative ESD were enrolled in this study. 45 cases infiltrated into the mucosal layer while 88 cases infiltrated into the submucosal layer. Postoperative pathology presented residual cancer in 18 (13.5%) patients and lymph node metastasis in 14 (10.5%) patients. There was neither residual tumor nor lymph node metastasis in 104 (78.2%) cases.

**Table 1:** Demographic and clinical features of patients

Characteristic	N = 133	Characteristic	N = 133
Age (y)		Lymphatic invasion	
≤60	46	Positive	17
>60	87	Negative	116
Gender (n)		Vascular invasion	
Male	94	Positive	17
Female	39	Negative	116
Tumor location		Perineural invasion	
Cardia and fundus	53	Positive	2
Body	27	Negative	131
Antrum	53	Horizontal margin	
Tumor size (cm)		Positive	11
≤2.0	58	Negative	122
>2.0	75	Vertical margin	
Tumor differentiation		Positive	21
Differentiated-type	67	Negative	112
Undifferentiated-type	12	Residual cancer	
Mixed-type	54	Positive	18
Tumor invasion		Negative	115
Mucosa (T1a)	45	Lymph node metastasis	
Submucosa (T1b)	88	Positive	14
Ulceration		Negative	119
Positive	17		
Negative	116		

### Risk factors of residual cancer in patients with non-curative ESD

The overall incidence of local residual cancer in patients who underwent additional surgery following non-curative ESD was 13.5% (18/133). In the univariate analyses, local residual cancer was correlated with histologic differentiation (differentiated type vs. undifferentiated type), and horizontal margin. Further multivariate analyses elucidated that horizontal margin (OR = 10.53, 95% CI: 2.59–42.83, P = 0.001) was an independent risk factor for local residual cancer (Table 2). For 18 patients with local residual cancer, we further analyzed the poor histoprognostic factors involved (Table 3). Among these 18 patients, 6 cases suffered positive horizontal margin, while 7 cases were mixed type differentiation. 6 cases had neither positive horizontal margin, nor mixed type differentiation.

**Table 2:** Univariate and multivariate analyses of risk factors for local residual cancer

			Univariate			Multivariate			
Characteristics		RC (%)	OR	95% CI	P	OR	95% CI		P
Age (y)									
≤60		8 (17.4)	1.000						
>60		10 (11.5)	0.617	0.225–1.690	0.347				
Gender (n)									
Male		14 (14.9)	1.000						
Female		4 (10.3)	0.653	0.201–2.125	0.479				
Tumor location					0.349				
Cardia and fundus		6 (11.3)	1.000	0.301–3.326	1.000				
Body		6 (22.2)	2.238	0.646–7.758	0.204				
Antrum		6 (11.3)	1.000						
Tumor size (cm)									
≤2.0		7 (12.1)	1.000						
>2.0		11 (14.7)	1.252	0.453–3.461	0.665				
Tumor differentiation					0.129			0.325	
Differentiated-type		7 (10.4)	1.000			1.000			
Undifferentiated-type		4 (33.3)	4.286	1.023–17.96	0.047	3.317	0.666–16.51	0.143	
Mixed-type		7 (13.0)	1.277	0.419–3.893	0.668	1.663	0.501–5.520	0.406	
Tumor invasion									
Mucosa (T1a)		9 (20.0)	1.000						
Submucosa (T1b)		9 (10.2)	0.456	0.167–1.244	0.125				
Ulceration									
Positive		2 (11.8)	0.833	0.174–3.993	0.820				
Negative		16 (13.8)	1.000						
Lymphatic invasion									
Positive		2 (11.8)	0.833	0.174–3.993	0.820				
Negative		16(13.8)	1.000						
Vascular invasion									
Positive		2 (11.8)	0.833	0.174–3.993	0.820				
Negative		16 (13.8)	1.000						
Perineural invasion									
Positive		0 (0.0)	0.000		0.999				
Negative		18 (13.7)	1.000						
Horizontal margin									
Positive		6 (54.5)	11.00	2.915–41.51	< 0.001	10.53	2.590–42.83	0.001	
Negative		12 (9.8)	1.000			1.000			
Vertical margin									
Positive		4 (19.0)	1.647	0.484–5.605	0.425				
Negative		14 (12.5)	1.000						

RC = residual cancer; OR = odds ratio; CI = confidence interval

**Table 3:** Patients with local residual cancer following additional surgery after non-curative endoscopic submucosal dissection

Case	Horizontal margin	Mixed-type differentiation
#1	-	-
#2	-	-
#3	-	-
#4	-	+
#5	+	-
#6	+	-
#7	+	-
#8	-	+
#9	-	+
#10	+	+
#11	-	-
#12	-	+
#13	+	-
#14	-	-
#15	-	+
#16	-	-
#17	-	+
#18	+	-

### Risk factors of lymph node metastasis in patients with non-curative ESD

The overall incidence of lymph node metastasis in patients who underwent additional surgery following non-curative ESD was 10.5% (14/133). As shown in Table 4, univariate analyses showed that lymph node metastasis was correlated with histologic differentiation (differentiated type vs. mixed type), lymphatic invasion, and vascular invasion. Further multivariate analyses elucidated that lymphatic invasion (OR = 8.797, 95% CI: 1.051–73.64, P = 0.045) was an independent risk factor for lymph node metastasis. For 14 patients with lymph node metastasis, we further analyzed the poor histoprognostic factors involved (Table 5). Among these 14 patients, 7 cases suffered lymphatic invasion, 6 cases suffered vascular invasion, and 10 cases were mixed type differentiation. Only one case had neither lymphatic invasion, vascular invasion, nor mixed type differentiation.

**Table 4:** Univariate and multivariate analyses of risk factors for lymph node metastasis

			Univariate			Multivariate			
Characteristics		LNM (%)	OR	95% CI	P	OR	95% CI		P
Age (y)									
≤60		8 (17.4)	1.000						
>60		6 (6.9)	0.823	0.571–1.186	0.296				
Gender (n)									
Male		8 (8.5)	1.000						
Female		6 (15.4)	1.955	0.630–6.063	0.246				
Tumor location					0.320				
Cardia and fundus		4 (7.5)	0.784	0.198–3.095	0.728				
Body		5 (18.5)	2.182	0.572–8.319	0.253				
Antrum		5 (9.4)	1.000						
Tumor size (cm)									
≤2.0		5 (8.6)	1.000						
>2.0		9 (12.0)	1.445	0.457–4.572	0.531				
Tumor differentiation					0.123				
Differentiated-type		4 (6.0)	1.000			1.000			
Undifferentiated-type		0 (0.0)	0.000		0.999	0.000		0.999	
Mixed-type		10 (18.5)	3.580	1.055–12.15	0.041	2.977	0.810–10.93	0.100	
Tumor invasion									
Mucosa (T1a)		4 (8.9)	1.000						
Submucosa (T1b sm1)		4(13.8%)	1.640	0.376–7.150	0.510				
Submucosa (T1b sm2)		6(10.2)	1.160	0.307–4.384	0.826				
Ulceration									
Positive		1 (5.9)	0.495	0.061–4.048	0.512				
Negative		13 (11.2)	1.000						
Lymphatic invasion									
Positive		7 (41.2)	10.90	3.181–37.35	< 0.001	8.797	1.051–73.64	0.045	
Negative		7 (6.0)	1.000			1.000			
Vascular invasion									
Positive		6 (35.3)	7.364	2.159–25.11	0.001	1.126	0.126–10.07	0.915	
Negative		8 (6.9)	1.000			1.000			
Perineural invasion									
Positive		0 (0.0)	0.000		0.999				
Negative		14 (10.7)	1.000						
Horizontal margin									
Positive		0 (0.0)	0.000		0.999				
Negative		14 (11.5)	1.000						
Vertical margin									
Positive		4 (19.0)	2.400	0.675–8.530	0.176				
Negative		10 (8.9)	1.000						

LNM = lymph node metastasis; OR = odds ratio; CI = confidence interval

**Table 5:** Patients with lymph node metastasis following additional surgery after non-curative endoscopic submucosal dissection

Case	Lymphatic invasion	Vascular invasion	Mixed-type differentiation
#1	+	+	+
#2	+	-	-
#3	-	-	-
#4	-	-	+
#5	-	-	+
#6	+	+	-
#7	-	-	+
#8	-	-	+
#9	+	+	+
#10	-	-	+
#11	-	-	+
#12	+	+	+
#13	+	+	-
#14	+	+	+

### Clinicopathologic features associated with different histologic types

Histopathologically, there were 67 (50.4%) cases with differentiated type, 12 (9.0%) cases with undifferentiated type, and 54 (40.6%) cases with mixed type (Table 1). The lymph node metastasis rate of differentiated type, undifferentiated type, and mixed type was 6% (4/67), 0% (0/12), and 18.5% (10/54), respectively (Table 4). Univariate analyses showed that histologic differentiation (differentiated type vs. mixed type) was correlated with lymph node metastasis (Table 4).

### Short-term and long-term outcomes of patients undergoing additional surgery after non-curative ESD

**Fig. 1:**
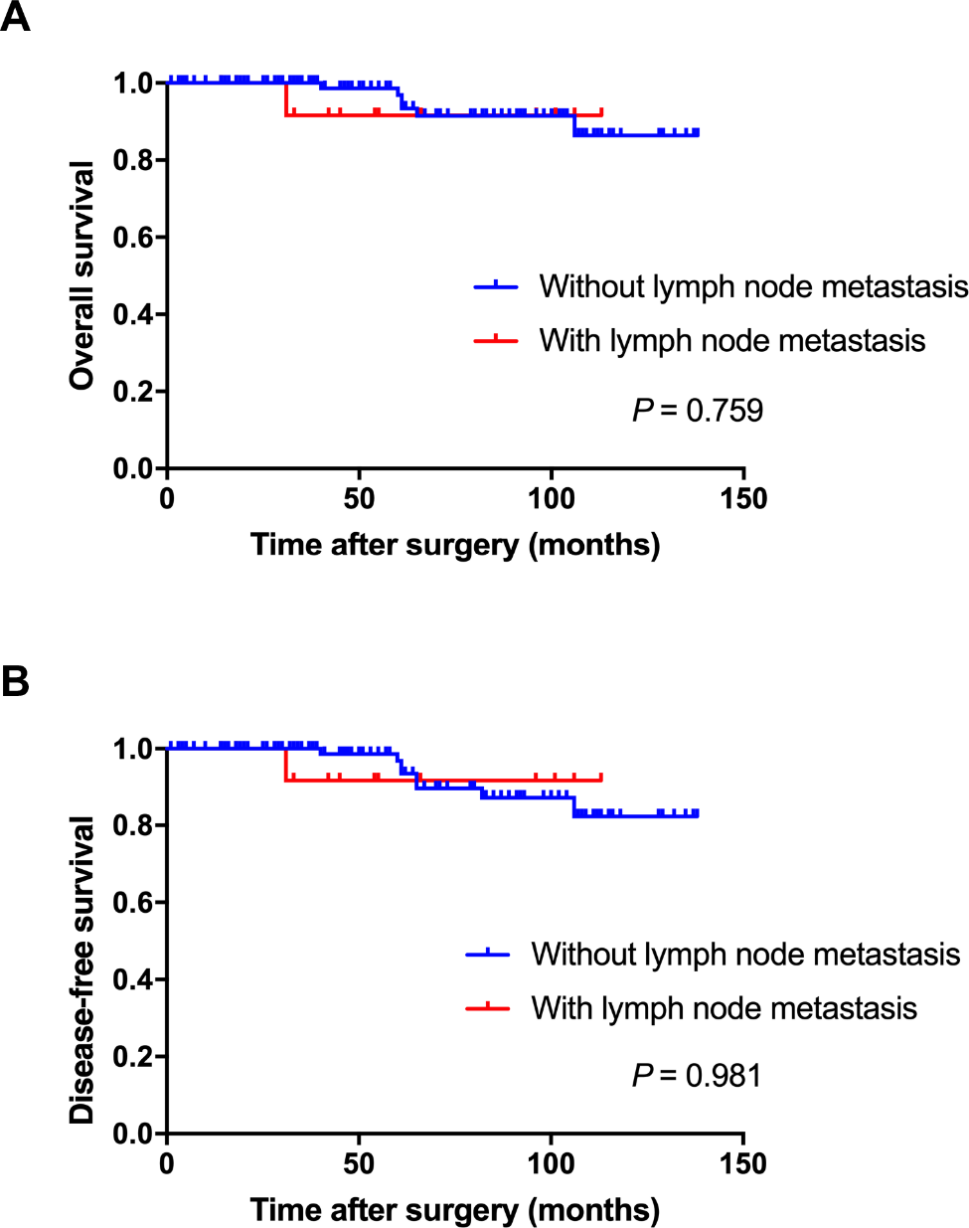
Overall survival and disease-free survival following additional surgery after non-curative endoscopic submucosal dissection for early gastric cancer

The short-term outcomes were evaluated based on the Clavien-Dindo (CD) classification system [5]. Of the 133 patients, 31 (22.3%) suffered postoperative short-term complications, and 4 (3.0%) suffered major complications (grade III or more). The details were presented in Table 6. The long-term outcomes were presented by Kaplan-Meier curves. The median follow-up period was 57 months. The overall survival and disease-free survival were shown in Fig. 1. No difference was detected between patients with or without lymph node metastasis both in overall survival (P = 0.759) and disease-free survival (P = 0.981).

**Table 6:** Short-term complications following additional surgery after non-curative endoscopic submucosal dissection for early gastric cancer

Characteristics	n	
Overall	31	
Grade I	13	
Fever > 37.5 °C	9	
Emesis	2	
Pleural effusion	2	
Grade II	15	
Blood transfusions	6	
Hypoalbuminemia	1	
Gastroparesis	1	
Wound infection	4	
Pneumonia	3	
Bacteremia	1	
Grade III	4	
Bleeding	3	
Pleural effusion	1	
Grade IV-V	0	

## Discussion

ESD was increasingly becoming a fundamental treatment for early gastric cancer because of its minimally invasive nature. Various large-scale clinical trials were carried out to expand the indications of endoscopic therapy. As more and more patients with early gastric cancer underwent endoscopic treatment, it was inevitable that more cases of non-curative resection will occur. In this case, a remedial gastrectomy with lymph node dissection would be recommended [6]. However, in approximately 90% of cases who underwent additional surgery, neither residual tumor nor lymph node metastasis was found [7]. This means that additional surgery might be over-medical for these cases. Therefore, it is necessary to screen out this subset of patients who benefit from additional surgery after non-curative ESD.

In our study, a total of 133 patients who underwent additional surgery after non-curative ESD were retrospectively analyzed. The incidence rates of residual cancer and lymph node metastasis were 13.5% (18/133) and 10.5% (14/133), respectively. There was neither residual tumor nor lymph node metastasis in 104 (78.2%) cases. Multivariate analyses elucidated that horizontal margin was an independent risk factor for local residual cancer, which was consistent with the previous studies [8–10]. Several studies showed that residual cancer was also correlated with positive vertical margin [11, 12]. However, we did not observe such a phenomenon. This might be attributable to a weaker cautery effect in the horizontal direction than that in the vertical direction [8]. Furthermore, the length of the positive margin might be more meaningful for predicting tumor residue. *Sangjeong et al.* reported that the sensitivity of a more than 6 mm positive margins length for predicting tumor residue was 85.7% [13]. As for differentiation type, undifferentiated type (compared with differentiated type) was significantly correlated with residual cancer in univariate analyses while not in multivariate analyses. An interesting finding was that none of the 11 cases with positive horizontal margins in our study was observed lymph node metastases in postoperative pathology. This means that for cases with positive horizontal margins and without lymphatic infiltration, repeat ESD might be optional.

Whether the lymph nodes metastasize was a critical factor in determining the treatment of early gastric cancer. In our study, the incidence rate of lymph node metastasis was 10.5% (14/133) in patients who underwent additional surgery after non-curative ESD. Multivariate analyses elucidated that lymphatic infiltration was an independent risk factor for lymph node metastasis. The proportion of lymph node metastasis was 6.9 times higher in patients with lymphatic infiltration than that without lymphatic infiltration. For 14 patients with lymph node metastasis, 7 cases suffered lymphatic invasion, 6 cases suffered vascular invasion, and 10 cases were mixed type differentiation. Only one case had neither lymphatic invasion, vascular invasion, nor mixed type differentiation. Previous studies revealed several other risk factors for lymph node metastasis in patients with non-curative ESD, including undifferentiated type, vascular infiltration, and positive vertical margin [14–16]. *Hatta et al.* constructed the eCura system to assess the risk of lymph node metastasis in patients after non-curative ESD [17]. This scoring system consisted of 5 factors: lymphatic infiltration, venous infiltration, positive vertical margin, SM2 infiltration, and tumor size > 3 cm. Our findings suggest that lymphatic infiltration appeared to play a more important role among these 5 factors. Therefore, follow-ups or repeat ESD might be sufficient for patients without lymphatic invasion.

Japanese Classification of Gastric Cancer divided early gastric cancer into differentiated and undifferentiated types [18]. In practice, some lesions comprised both differentiated and undifferentiated types. Recent studies revealed that the incidence rate of lymph node metastasis was higher in patients with mixed histologic type than differentiated type or even undifferentiated type [19, 20]. From tumorigenesis to clinical features, the mixed histologic type was different from the pure histologic type. Therefore, some arguments suggested that mixed histologic type should be treated as a separate subtype [21]. The 5th edition JGCA guidelines determined whether the criteria for curative resection were met based on the size and depth of invasion of undifferentiated components in mixed histologic type EGC [1]. In this study, we considered the mixed histologic type as a separate subtype. The proportion of mixed histologic type was 40.6% (54/133) in patients who underwent additional surgery after non-curative ESD. In contrast, our previous study elucidated that the proportion of mixed histologic type was 27.7% (202/730) in all early gastric cancer who underwent radical gastric resection [22]. The above results implied that patients with mixed types were more likely to undergo additional surgery after non-curative ESD. Moreover, for patients undergoing additional surgery after non-curative ESD, the lymph node metastasis rate of differentiated type, undifferentiated type, and mixed type was 6% (4/67), 0% (0/120), and 18.5% (10/54), respectively. Univariate analyses showed that histologic differentiation (differentiated type vs. mixed type) was correlated with lymph node metastasis. Mechanistically, former studies have made some possible explanations for why mixed type GC were more aggressive than pure type GC. These hypotheses involved genetic and epigenetic abnormalities, interactions with the tumor microenvironment, and intratumor evolution [23–25]. *Park et al.* disclosed that CpG island promoter hypermethylation was higher in mixed type GC than pure type GC [26]. *Sentani* and colleagues reported that mixed type GC showed a characteristically expression of cancer stem cell-related molecules (CD44, CD133, and ALDH1), receptor tyrosine kinase molecules (EGFR, c-MET, and HER2), and chromosomal instability compared to pure type GC [27]. Thus, it is meaningful to diagnose mixed type early gastric cancer before ESD procedures, which might reduce the incidence rates of additional surgery after ESD caused by incorrect pretreatment diagnosis of histological type. Magnifying endoscopy combined with narrow-band imaging and biopsy was a promising measure for diagnosing mixed histologic type EGC [28, 29].

We acknowledged some limitations in our study. First, it was a retrospective study. The sample size was relatively small from a single center. Second, the indications for ESD have been expanding in recent years. There might be a selection bias for the criteria of ESD between different endoscopists. Third, only surgical cases were enrolled in this study, some patients who underwent follow-ups without additional surgery have not been recorded. Further study is needed to compare the prognosis between patients with follow-ups or additional surgery.

## Conclusion

In conclusion, for patients who underwent non-curative ESD, positive horizontal margin was an independent risk factor for residual cancer, while lymphatic infiltration was an independent risk factor for lymph node metastasis. Early gastric cancer with mixed histologic type might have a higher rate of lymph node metastasis. Therefore, more attention should be paid to mixed histologic type when developing criteria for EDS resection.

### Electronic supplementary material

Below is the link to the electronic supplementary material.

Supplementary Material 1

## Data Availability

The datasets analyzed during the current study are available from the corresponding author on reasonable request.

## Abbreviations

EGC: Early gastric cancer
EMR: Endoscopic mucosal resection
ESD: Endoscopic submucosal dissection
