# Supplementary material for: Risk factors of additional surgery after non-curative endoscopic submucosal dissection for early gastric cancer
Source: BMC Gastroenterol. 2023 Nov 10;23:383. doi: 10.1186/s12876-023-03006-9 (PMC10636959; doi:10.1186/s12876-023-03006-9)
Supplement: Supplementary file 1 — Supplementary Material 1 [file 12876_2023_3006_MOESM1_ESM.docx]

**Supplemental Table 1. Evaluation of endoscopic curability.**

| Depth of invasion | Ulceration | Differentiated-type | | Undifferentiated-type | |
| --- | --- | --- | --- | --- | --- |
| pT1a (M) | UL0 |  |  | ≤2cm | >2cm |
|  |  |  |  |  |  |
|  | UL1 | ≤3cm | >3cm |  |  |
|  |  |  |  |  |  |
| pT1b1 (SM1) | | ≤3cm | >3cm |  |  |
|  |  |  |  |  |  |
| pT1b2 (SM2) | |  |  |  |  |
|  |  |  |  |  |  |

|  |
| --- |

|  |
| --- |

|  |
| --- |

eCuraA* eCuraB* eCuraC-2

*, Confined to en bloc resection and HM0, VM0, Ly0, and V0. pT1a (M), intramucosal cancer (histopathological diagnosis); pT1b (SM), submucosally invasive cancer (histopathological diagnosis). UL, finding of ulceration (or ulcer scar); UL0, absence of ulceration or ulcer scar; UL1, presence of ulceration or ulcer scar; HM, Horizontal margin; VM: Vertical margin; Ly, Lymphatic invasion; V, Vascular invasion.
